# Involvement of Human Papillomaviruses in Cervical Cancer

**DOI:** 10.3389/fmicb.2018.02896

**Published:** 2018-11-28

**Authors:** Xuelian Wang, Xiumin Huang, Youzhong Zhang

**Affiliations:** ^1^Department of Gynecology and Obstetrics, Qilu Hospital of Shandong University, Jinan, China; ^2^Department of Gynecology and Obstetrics, Zhongshan Hospital of Xiamen University, Xiamen, China

**Keywords:** human papillomaviruses, cervical cancer, HPV-induced carcinogenesis, HPV genotype, HPV vaccine

## Abstract

Human papillomaviruses (HPV) are the first viruses to have been acknowledged to prompt carcinogenesis, and they are linked with cancers of the uterine cervix, anogenital tumors, and head and neck malignancies. This paper examines the structure and primary genomic attributes of HPV and highlights the clinical participation of the primary HPV serotypes, focusing on the roles that HPV-16 and 18 play in carcinogenesis. The mechanisms that take place in the progression of cervical neoplasia are described. The oncogenic proteins E6 and E7 disrupt control of the cell cycle by their communication with p53 and retinoblastoma protein. Epidemiological factors, diagnostic tools, and management of the disease are examined in this manuscript, as are the vaccines currently marketed to protect against viral infection. We offer insights into ongoing research on the roles that oxidative stress and microRNAs play in cervical carcinogenesis since such studies may lead to novel methods of diagnosis and treatment. Several of these topics are surfacing as being critical for future study. One particular area of importance is the study of the mechanisms involved in the modulation of infection and cancer development at cervical sites. HPV-induced cancers may be vulnerable to immune therapy, offering the chance to treat advanced cervical disease. We propose that oxidative stress, mRNA, and the mechanisms of HPV infection will be critical points for HPV cancer research over the next decade.

## Introduction

Human papillomaviruses (HPVs) are minute viruses that carry deoxyribonucleic acid (DNA) and are part of the Papillomaviridae family. They are ubiquitous and able to adjust to their hosts. They have the ability to hide efficiently from immune reactions. More than 200 types of HPV have been established and classified into 29 genera, and the majority of them impact humans. These viruses mainly target differentiating squamous epithelium, and they are linked to cutaneous infections, affecting nearly every part of human skin and causing mucosal infections. HPV infection is a risk factor for malignancy of the uterine cervix as it has a pivotal role in carcinogenesis via the activation of its genomic products (Cubie, 2013).

### HPV Genome

The genome of all papillomaviruses has 3 dissimilar areas, known as the upstream regulatory region (URR), the first area, and the second area. The URR is also known as the long control region (LCR) or the non-coding region (NCR) and comprises about 10% of the complete genome (IARC, 1995). The first area fills about half of the genome and is split into two large and several small reading frames: E1–E2 and E4–E7 are the large reading frames (IARC, 1995), and E6 and E7 contain the small reading frames, which participate in the progression of cervical cancer (Cubie et al., 2012). The second area fills the rest (40%) of the genome and contains the genes L1 and L2 (IARC, 1995).

### HPV Classification

While HPV is frequently linked to incidents of cervical cancer, it is not the only medical condition with which the virus has been associated. There are many HPVs that induce different lesions, and they can cause overlapping outcomes. HPV serotypes are genetically different from each other, and the typical classification system indicates that a certain HPV type has to have a full genome in which the L1 nucleotide sequence varies from that in any other HPV genome by at least 10%. HPV types are assigned numerically in chronological order based on the date of their discovery (Cubie et al., 2012).

**FIGURE 1:**
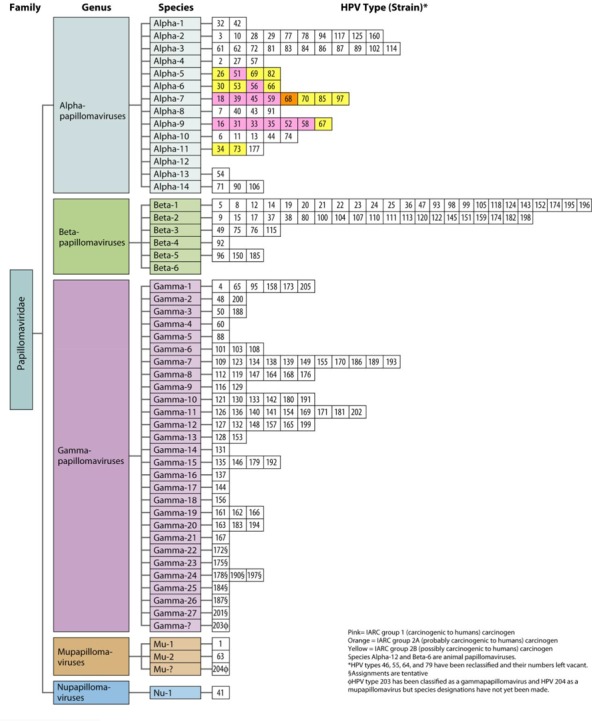
HPV classification based on the nucleotide sequence of the capsid protein L1 gene (Burd, 2016).

There are currently 39 genera in the family Papillomaviridae. The HPVs are contained in five of those genera (alphapapillomaviruses, betapapillomaviruses, gammapapillomaviruses, mupapillomaviruses, and nupapillomaviruses) (Figure 1). In 2012, all the different HPVs were established and designated as either group 1 (carcinogenic to humans) carcinogens, group 2A carcinogens (probably carcinogenic to humans), or group 2B carcinogens (possibly carcinogenic to humans) by the International Agency for Research on Cancer (IARC) (Figure 1). The group-1 HPVs (HPV16, HPV18, HPV31, HPV33, HPV35, HPV39, HPV45, HPV51, HPV52, HPV56, HPV58, and HPV59) are considered to be hazardous in high-pressure liquid chromatography. There is little research suggesting HPV68 as high risk, so it is placed in group 2A as probably carcinogenic. Of all of the cervical cancers, 96% can be linked to one of the 13 HPV types in groups 1 and 2A (Arbyn et al., 2014). Additional alpha papillomaviruses (HPV26, HPV30, HPV34, HPV53, HPV66, HPV67, HPV 69, HPV70, HPV73, HPV82, HPV85, and HPV97) have been linked to infrequent incidences of cervical cancer and are thought to be group 2B carcinogens. Because fewer cases of cervical cancer are associated with the group 2B HPVs, it is more difficult to assess their carcinogenicity. Nevertheless, research has shown that markers of HPV-induced carcinogenesis, including E6 mRNA, elevated the expression of p16 and reduced the expression of cyclin D1, p53, and Rb. This pattern has been observed in cervical cancers related to all of the HPV carcinogen groups (Arbyn et al., 2014). If the 2.6% of cervical cancer cases linked to group 2B HPV-type carcinogens are grouped with the 96% of cases attributable to group 1 and 2a type carcinogens, a total of 98.7% of all cervical cancers are HPV-positive cancers. Additional data have demonstrated that HPV68, HPV26, HPV66, HPV67, HPV73, and HPV82, although rare, are found with greater frequency in women with cervical cancer than in those with normal cervical cytology; therefore, an update to the carcinogen classification system should be considered (Arbyn et al., 2014; Halec et al., 2014).

## Epidemiology

**FIGURE 2:**
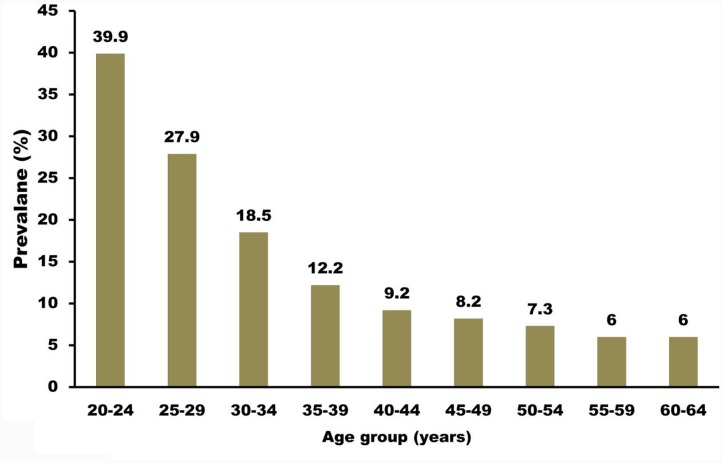
Prevalence of high-risk HPV infection among females undergoing cervical screening. HPV testing in routine cervical screening: cross sectional data from the ARTISTIC trial (Kitchener et al., 2006). Compiled using information from Kitchener et al. (2006).

Anogenital infection is the most frequent sexually transmitted infection (STI) in the United States; approximately 6.2 million people (most frequently adolescents and young adults) are afflicted with this type of infection annually. Clinical evaluations have demonstrated that the occurrence of STI in adolescent girls is typically about 30% and can escalate to 64% in certain populations. A separate evaluation stated that at 4 years after their first sexual intercourse experience, more than 50% of the young female population was afflicted with a cervical HPV infection (Brianti et al., 2017). The same study also documented that HPV may be transmitted via non-penetrative sexual behavior, but the probability of this is lower than that from obtaining the infection via penetrative intercourse. Aside from sexual history, evaluations in the United States have documented that patients aged younger than 25 years are also at risk of infection. The occurrence seems lower after this age in most studies, with the exception of one cohort study in Costa Rica that discovered that the occurrence increases once again after 40 years of age. In addition, men and women appear to have close HPV infection rates. Figure 2 shows the occurrence of HPV infection based on patient age.

The identification of high-risk HPV in virgins, neonates, and children offers proof that sexual interaction is not the only method of HPV transmission. Low- and high-risk HPV serotypes can be circulated through non-sexual methods, including touching others, communal bathing, and touching contaminated fomites (Brianti et al., 2017). It seems that the transfer of high-risk HPV from mother to infant occurs during parturition as the neonate goes down the infected birth canal. This more frequently happens in mothers with exceptionally large amounts of HPV DNA, especially in those with HPV-16, than in those with less DNA. Moreover, there has been a report of two mothers with both HPV-16 and 18 infections who gave birth to neonates with identical co-infections (Cason et al., 1998).

Pregnancy appears to escalate the chance of contracting genital HPV infections. This was shown in a study in which 52.5% of mothers tested positive for HPV DNA in the third trimester of pregnancy, as opposed to 17.5% after parturition (Brianti et al., 2017). A physiological explanation for this could be that the hormone profile in pregnancy escalates the transcription of HPV genes because of the communication between glucocorticoids and their response elements in the non-coding area of HPV-16. Furthermore, pregnant women are in a state of immunosuppression. HPV infections can also be transferred throughout pregnancy through the placenta and amniotic fluid. One study demonstrated that HPV DNA was in 75% of amniotic fluids obtained from mothers who tested positive for cervical HPV DNA (Cason et al., 1998).

Worldwide, cervical cancer is the second most common malignancy in women, impacting about 35 of every 100,000 women (Cubie, 2013). Attention given to the link between HPV and cancer was significantly raised when HPV types 16 and 18 were detected in cervical cancers and preneoplastic dysplasia, the lesions that can make a woman susceptible to malignancy of the uterine cervix. HPV DNA has been determined to be present in more than 99% of cervical cancer cases; however, the most frequent high-risk serotypes are different among countries, ethnicities, and socioeconomic statuses. In a study featuring over 30,000 cervical cancers, IARC showed that of the most frequent HPV serotypes that lead to cervical malignancy (16, 18, 58, 33, 45, 31, 52, 35, 59, 39, 51, and 56), HPV 16 induces more than 50% of cervical cancers, while HPV 16 and 18 together lead to over 70% of cases across the globe (Burd, 2016). HPV serotypes 18 and 45 are implicated in the more aggressive cervical adenocarcinomas (Cubie, 2013).

It has been discovered that long-term infection with high-risk HPV serotypes is the greatest risk factor in the progression from precursor lesions to cervical cancer. Persistence is typically defined as the identification of identical high-risk HPV types at >2 visits that are separated by 4–6 months (Dunne and Markowitz, 2006). In fact, studies have shown that such persistent infections can increase the likelihood of developing high-grade precursors of cervical malignancy more than tenfold (Dunne and Markowitz, 2006).

## Pathophysiology

### Cervical Canal and Associated Malignancies

The cervix, which is located between the vagina and the uterus, is a canal with two openings: the superior internal os that goes into the uterus and the inferior external os that goes into the vaginal cavity. The histology of the cervical canal is characterized by simple columnar secretory epithelium, as opposed to the vaginal cavity, which is lined by stratified non-keratinizing squamous epithelium. The epithelia that line the endocervix and exocervix join at the transition zone, or squamocolumnar junction, correlating to the area of the cervix at the external os (Livolsi, 2002).

The squamocolumnar junction is a crucial cytological landmark since it is the area that is the most vulnerable to HPV infection, and it is the place in which over 90% of lower genital tract malignancies initiate (Burd, 2016). HPV is recognized as inducing cervical dysplasia and cervical intraepithelial neoplasia (CIN), which typically develop into cervical cancer due to an ongoing infection with high-risk HPV (Narisawasaito and Kiyono, 2007).

Since the transition zone includes two kinds of epithelial cells (glandular and squamous cells), two different forms of cancers can occur in the cervix. An unregulated rapid increase of glandular cells in the endocervix generates an adenocarcinoma in 10–20% of cases, although the incidence seems to be on the rise in recent years (Brianti et al., 2017). A squamous cell malignancy is the cause of squamous cell carcinoma. The latter is much more frequent (occurring in 80–90% of cases) and is typically asymptomatic in its first stages, but can cause coital and pelvic pain and deviant vaginal bleeding and discharge as it progresses (Brianti et al., 2017).

### Life Cycle of HPV

**FIGURE 3:**
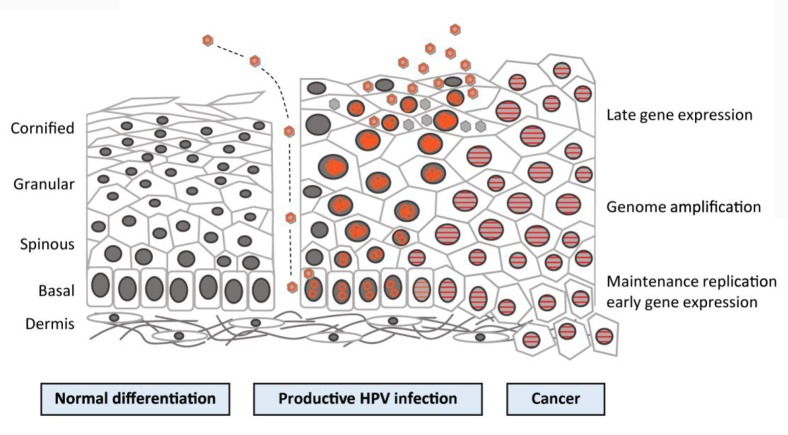
Human papillomavirus (HPV) life cycle and cancer. Cartoon depicting normal stratified cervical epithelium (left), HPV-infected epithelium (center), and HPV-induced cancer (right). Epithelial layers are indicated on the far left, and HPV life cycle stages are indicated on the far right. Episomal genomes are shown as orange circles and integrated genomes are shown as orange stripes. (Left) Normal keratinocyte differentiation: basal cells divide and daughter cells migrate upward, beginning the differentiation process. As differentiation proceeds, cells exit the cell cycle. Fully keratinized squames slough off from the apical surface. (Middle) Productive HPV infection: HPV virions gain access to basal cells via microwounds. The viral genomes migrate to the nucleus, where they are maintained at ˜100 copies/cell. As daughter cells begin differentiation, viral genomes are amplified. Cell nuclei are retained and chromatin is activated to support viral DNA replication. (Right) Cancer: viral genomes often integrate into the host genome and E6/E7 expression is increased, leading to enhanced proliferation and the accumulation of cellular mutations. Cellular differentiation is lost, and cancerous cells invade into the dermal layer as well as into neighboring tissues (Langsfeld and Laimin, 2016).

Cervical carcinogenesis is strongly associated with the events that happen in the life cycle of the virus, as shown in Figure 3. In a stratified squamous epithelium, the cells creating the basal layer act as stem cells, and thus they undergo cell division when they replace the cells released from the surface layer. When a basal cell divides via mitosis, two daughter cells are created: one rises and changes into a terminally differentiated cell and the other cell stays in the basal layer to retain the pool of dividing cells. The initial targets of the virus are the basal cells that are vulnerable via microwounds. HPV virions proceed into the cells by interacting with certain receptors, such as alpha-6 integrin, which binds HPV-16 (Narisawasaito and Kiyono, 2007). Viral DNA replication starts in the basal layers, generating 50–100 copies of the genome in every cell. This is followed by the expression of the E1 and E2 proteins that are required for the replication procedure and for the separation of recently synthesized DNA, therefore guaranteeing that infected stem cells stay in the lesion for an extended period of time. The virus mostly uses host equipment to perform DNA replication, with the exception of the E1 helicase (Narisawasaito and Kiyono, 2007). Early gene products, such as E5–E7, are believed to produce a favorable environment for replication to take place by encouraging DNA replication in the host cell and halting apoptosis (Narisawasaito and Kiyono, 2007).

As the infected basal cells move up and differentiate, the viral late genes L1 and L2 are transcribed, thus prompting the vegetative stage of the life cycle distinguished by dramatic amplification of the genome (Narisawasaito and Kiyono, 2007). It appears that control over the expression of late genes depends on the state of differentiation of the host cell (Howley, 2006). Once the cell reaches the outermost layer of the epithelium, the newly synthesized viral DNA is encapsulated to form new virions, which are released, and the life cycle is then repeated. As HPVs do not induce complete lysis in host cells, new virions are deposited in squames that are continuously shed (Hikita and Kozako, 2001). It is intriguing that the virus is greatly hidden from the host immune system, since the immunogenic virions are put together only in the outer portions of the epithelium. In addition, viral proteins E6 and E7 act to ensure that the infection remains asymptomatic by deactivating interferon regulatory factor (Narisawasaito and Kiyono, 2007).

Squamous epithelial cells infected by HPV undergo koilocytosis to become cells called koilocytes. Compared to normal cells, these cells have a larger, darker, and asymmetrically outlined nucleus encompassed by an area of transparent space, termed a perinuclear halo, and they appear to be vacuolated. This alteration suggests minor cellular dysplasia and shows a highly replicative viral state. When the dysplasia is moderate or severe, the cells are small and multiply on the uppermost portion of the epithelium, creating a potentially carcinogenic lesion if it is severe (Camilleri and Bundell, 2013).

### Role of HPV in Cervical Carcinogenesis

While HPV is the greatest risk factor for cervical cancer, many researchers propose that the specific integration of viral DNA in the host cell does not frequently happen, and in the majority of the time, HPV infection is removed quite speedily by the immune system. While viral DNA can lead to fast neoplastic alteration of infected cells once it is incorporated, the existence of HPV DNA in the cell by itself is not enough to induce cancer, as additional genetic and epigenetic occurrences are likely needed (Narisawasaito and Kiyono, 2007).

Two main oncogenic protein products of the HPV virus are E6 and E7; they act by modifying the control of the cell cycle and by regulating apoptosis. The incorporation of viral DNA disrupts the activity of the E2 protein. The E2 protein is recognized as having the ability to repress the transcription of E6 and E7, and thus its interruption causes dysregulated expression of these oncoproteins. Combined, these proteins are able to immortalize cells, so that cells retain their mitotic ability to generate clones that also have the immortalized phenotype and do not experience terminal differentiation (Howley, 2006). The immune response is a key factor in the fight against HPV infection and cervical carcinogenesis. However, HPV is able to promote immune evasion through the expression of the E5 oncogene, which is responsible for modulation of several immune mechanisms, including antigen presentation and inflammatory pathways (de Freitas et al., 2017).

### E7 Oncoprotein

The major characteristic of E7 that enables it to enhance neoplastic change is its interaction and subsequent inactivation of pRb. The phosphorylation state of pRb is modified based on the phase of the cell cycle, as it is dephosphorylated in G0 and G1 phases. It is phosphorylated during the S-phase and stays phosphorylated until later in the M-phase when it takes on a hypophosphorylated appearance again due to the action of a certain phosphatase (Howley, 2006).

When dephosphorylated, pRb and its associated proteins inhibit transcription factors, such as E2F, by binding to them, thereby repressing the expression of genes whose products stimulate DNA synthesis and enhancing the progression of the cell cycle. In contrast, when pRb is phosphorylated by G1 cyclin D kinases (CDKs), it cannot bind to E2F and its inhibitory impact is thereby removed, enabling the cell to move to the S-phase. Thus, it is a regulator of the G1/S checkpoint. The identification of damaged DNA results in the activation of p53, which then initiates p21, a CDK inhibitor. This p21 links to and limits cyclin E-CDK2. Thus, pRb cannot be phosphorylated. As a result, pRb can inhibit E2F and halt the G1/S transition (Shackelford et al., 1999).

The E7 protein can link to the hypophosphorylated form of pRb, thus disturbing the complex generated between pRb and E2F. This causes an early movement of the cell into the S-phase, thus leading to DNA synthesis and, lastly, cell division. Interestingly, the actual production of E7 and its effects on targets that include pRb are necessary for the replication and completion of the full life cycle of HPV (Hikita and Kozako, 2001).

The E7 oncoprotein was observed to modulate the DNA methylation mechanism to control pathways of cellular propagation, and may bring about epigenetic changes through the Rb family of tumor suppressor proteins (Sen et al., 2018). A study showed that DNA methyl transferase DNMT1 could be linked by HPV-16 E7 both *in vitro* and *in vivo* to initiate its enzymatic actions (Yin et al., 2016). The E7 oncoprotein can directly bind to DNMT1 and induce gene silencing by hypermethylation (Sen et al., 2018). E7 can form a tight complex with Rb resulting in release of E2F, which then binds to DNMT1, causing hypermethylation of CpG islands (Duenas-Gonzalez et al., 2005).

### E6 Oncoprotein

The E6 protein mainly shows its neoplastic impact on HPV-infected cells by encouraging the ubiquitin-dependent proteosomal degradation of p53 (Narisawasaito and Kiyono, 2007), a tumor suppressor gene product that prevents the buildup of destructive mutations that can cause cancer to develop. Such mutations can be due to DNA damage by physical and chemical mutagens, as well as errors that occur during DNA replication. Upon the identification of abnormal DNA and p53 activation, the cell cycle is halted, enabling DNA repair to happen prior to the cell splitting. In particular situations, including when the DNA cannot be repaired, apoptosis can be initiated for programmed cell death (Hikita and Kozako, 2001).

The concentration of p53 in cells with E6, including cervical cancer cells, is about 2–3 times lower than in healthy cells. Its half-life is also substantially decreased. As a result, the typical response of p53 to DNA damage does not occur. DNA mutations remain in the genome unrepaired and are carried from one cellular generation to the next, ending up with a buildup over time leading to genomic fluctuations (Howley, 2006). Thus, apart from the lack of checkpoint surveillance for DNA damage in cancer cells, these cells also have an intrinsic tendency to favor mutagenesis (Hikita and Kozako, 2001).

The binding of E6 to p53 is not automatic; it is regulated by E6-associated protein (E6AP), an E3 ubiquitin protein ligase. E6AP is in a group of proteins similar to the E6-AP carboxyl terminus (HECT) E3 ligases that act in the identification of substrates via ubiquitylation machinery aimed at proteosomal degradation. Interestingly, the presence of E6 increases the turnover of E6AP, probably as a result of its enhanced enzymatic activity in the HPV-infected cellular environment (Howley, 2006).

The mechanism of E6 mediated gene silencing has been reported (Sen et al., 2018). The mechanism involves degradation of p53 and release of specificity protein 1 (Sp1) transcription activator, which binds to the promoter of DNMT1 and upregulates the expression of this gene. Then, the elevated amount of DNMT1 leads to hypermethylation of DNA.

### E5 Oncoprotein

E5 was proposed to be classified as a viroporin, a channel protein able to modulate ion homeostasis, vesicle trafficking, virion production, and viral genome entry (Wetherill et al., 2012). In HPV16 infected cells, E5 oncoprotein plays a key role in cell growth and impairs several signal transduction pathways. Furthermore, pro-carcinogenic activities are also performed by HPV16 E5, including the stimulation of EGF-mediated cell proliferation, the inhibition of apoptosis induced by tumor necrosis factor ligand (TNFL) and CD95 ligand (CD95L) (de Freitas et al., 2017), and the modulation of genes involved in cell adhesion and cell motility (Kivi et al., 2008). All of these are activities that indirectly intervene in the host’s immune system.

### Immune Avoidance in HPV Infection, Squamous Intraepithelial Lesions (SIL), and Cervical Cancer

Human papillomaviruses has a few mechanisms to evade the immune system: it downregulates interferon expression and upregulates interleukin (IL)-10 and transforming growth factor (TGF)-β1, producing a local immunosuppressive environment; along with altered tumor surface antigens, this environment establishes an immunosuppressive network that blocks the antitumor immune response (Torres-Poveda et al., 2014). In patients with high-risk HPV infections of the cervix and with SIL, the presence of IL-10 and TGF-β1 might initially create conditions that encourage an immunosuppressive microenvironment in the lesion, which could negatively affect the cellular immune response (Sasagawa et al., 2012; Torres-Poveda et al., 2014). Such a microenvironment can encourage the persistence of viruses and lead to cervical cancer (Stanley et al., 2007). In serum and cervical tissues from patients with high-risk HPV infections, low-grade SIL, high-grade SIL, and cervical cancer (Giannini et al., 1998; Stanley et al., 2007), the cytokines IL-10 and TGF-β1 have been detected. The levels of these cytokines are correlated with lesion severity (Bermudez-Morales et al., 2008). A previous review indicated that local immunosuppression distinguishes cervical cancer, and this immunosuppression is dependent on Th2/Th3 cytokines. These data accord with cervical biopsy findings in which there is a pattern of Th2/Th3 cytokine expression in cervical cancer tissues that is non-existent in the normal cervix, indicating that HPV infection initiates immunosuppressive cytokine transcription to avoid eliciting a response from the host immune system (Sheu et al., 2001; Alcocer-Gonzalez et al., 2006).

Interleukin-10, a powerful immunosuppressive cytokine, and TGF-β1 promote each other’s expression. They also promote the production of HPV-16 E6 and E7 proteins, which induce the TGF-β1 and IL-10 genes, establishing a vicious cycle (Peralta-Zaragoza et al., 2006). CD3 expression is downregulated by IL-10 and TGF-β1, and this has a crucial role in the activation of T-cells. Lastly, IL-10 and TGF-β1 enlist Treg cells, which cause serious peripheral tolerance. Therefore, we hypothesize that IL-10 and TGF-β1 promote immune system avoidance via establishment of an immunosuppressive state in the cervixes of HPV-infected women. These findings will be especially pertinent to HPV vaccine production and the development of novel targeted immunotherapies for women who have LGSIL, HGSIL, and cervical cancer (Peralta-Zaragoza et al., 2012).

As research on the immune system and HPV-associated cervical cancer progresses, there are more questions than answers. Researchers have shown that T-lymphocytes isolated from patients with cervical lesions and cervical cancer are only partially activated and that there are lowered expression levels of a few signal transduction molecules that take part in the full activation of T-lymphocytes. Therefore, it is of special interest to evaluate if type Th1 cytokines can counteract the expression of these molecules and if researchers can produce completely functional HPV-specific T-lymphocytes.

### Areas of Current Research

**FIGURE 4:**
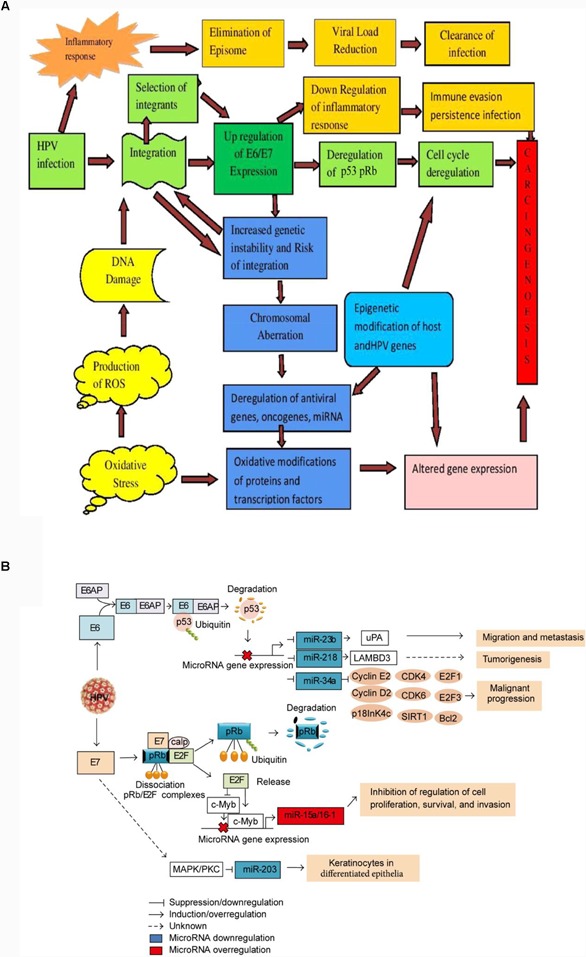
Schematic model of HPV-driven carcinogenesis. **(A)** A multistep molecular mechanism of host-viral interaction (Senapati et al., 2016). The initial outcome of carcinogenesis is modulated by both viral (high-risk versus low-risk HPV types, HPV integration) and host factors (inflammatory response, oxidative stress). Inflammatory response upon initial infection such as IFN response plays role in reducing episomal HPV resulting clearance of infection. Integration of HPV is *(initiated with DNA damage. The IFN induced loss of episomal HPV and down-regulation of E2 leads to the selection of cells with integrated HPV genomes expressing higher levels of E6 and E7. Once the early genes E6 and E7 are expressed, TLR9 down regulated and IFN response impaired, resulting a conducive milieu for immune evasion and persistent infection. Up regulation of E6/E7 increases genetic instability and chromosomal rearrangements that increase the risk of integration. Overexpression of E6/E7 leads to deregulation of the cell cycle via p53 and Rb degradation, deregulation of oncogenes and miRNAs expression. Epigenetic and genetic modification in viral and host genome leads to the deregulation of E6 and E7 oncogenes, and host tumor suppressor genes that lead to carcinogenesis. Oxidative modification of TFs also leads to altered gene expression and carcinogenesis. **(B)** Schematic model of the interaction between microRNAs and factors involved in malignant transformation caused by HPVE6 and E7 expression in cervical cancer cell (del Mar Diaz-Gonzalez et al., 2015). E6 disrupts the expression of miR-23b, miR-218, and miR-34a via p53 degradation and their expression is transactivated by the binding of p53 to consensus sites in the promoter regions, affecting the expression of cell cycle regulators, such as E2, cyclin D1, CDK4, CDK6, E2F1, E2F3, E2F5, Bcl-2, SIRT1, p18, uPA, and LAMBD3. In the overexpression of miR-15/16 cluster byE7, E2F1 transactivates the c-Myb expression and represses the c-Myc expression, and then the microRNA cluster regulation is controlled by binding of c-Myc or c-Myb to promoter region of microRNA cluster. The increased expression of miR-15a/miR-16-1 induces the inhibition of cell proliferation, survival, and invasion. The down regulation of miR-203 by E7 is mediated by MAPK/PKC pathway.)*

Research is now closing in on the role oxidative stress plays in HPV-mediated carcinogenesis. Reactive oxygen species prompt DNA damage and regulate the viral life cycle. Evaluations have suggested a link between the oxidative status of infected cells and their prolonged existence or further development of lesions. Reactive oxygen species also have pro-survival and anti-apoptotic effects on infected cells, and they increases the expression of the E6 and E7 genes (Figure 4A). This seems to be an encouraging area to examine, in particular to establish the impacts of oxidative stress on chemotherapy reactions and resistance and the prospective use of markers of oxidative stress for diagnostic purposes (Marco, 2013).

Micro-RNAs (miRNAs), which are short strands of non-coding RNA that normally have a short half-life and alter gene expression post-transcriptionally, appear to have a role in cervical carcinogenesis. Their deregulation is linked with the initiation, development, and spread of human tumors, such as cervical cancer, which shows elevated and lowered levels of oncogenic and tumor-suppressive miRNAs, respectively. The E6 and E7 proteins can regulate miRNAs (Figure 4B). Thus, it is helpful to establish exactly how they will be utilized as prognostic biomarkers by contrasting the miRNA profiles of healthy and modified cells. Further, additional studies may potentially discover another type of cervical cancer treatment using RNA modification therapy (Gómez-Gómez et al., 2013).

The E6 and E7 oncoproteins interact with and/or modulate the expression of many proteins involved in epigenetic regulation, including DNA methyltransferases, histone modifying enzymes, and subunits of chromatin remodeling complexes, thereby influencing the host cell transcription program. Furthermore, HPV oncoproteins modulate expression of cellular micro RNAs. Most of these epigenetic actions participate in a complex dynamic interplay involved in the maintenance of persistent infection, cell transformation, and development of invasive cancer by a considerable deregulation of tumor suppressor proteins and oncogenes (Durzynska et al., 2017; Sen et al., 2018).

Recently, a detailed analysis of the Cancer Genome Atlas (TCGA) cervical cancer gene expression, DNA methylation, and somatic mutation profile was reported (Banister et al., 2017). A subset of tumors, which no longer express HPV E6/E7 oncogenes (HPV-inactive), were identified. These tumors have gene expression, DNA methylation and somatic mutation signatures different from HPV-active tumors, and are more similar to other, viral-independent, cancers. Implications for cervical cancer progression and opportunities for targeted therapy have been discussed (Banister et al., 2017).

Autophagy is the physiological cellular route that accounts for removal, degradation, and recycling of damaged organelles, proteins, and lipids in lysosomal vacuoles. In addition to this scavenger function, autophagy plays a fundamental role during viral infections and cancers and is, therefore, frequently exploited by viruses to their own benefit. Recently, a link between HPV and autophagy has clearly emerged, leading to a possibility for development of novel anti-viral strategies aimed at restraining HPV infectivity (Mattoscio et al., 2018). How the oncogenic HPV16 virus can usurp autophagy has been described, including highlighting similarities and differences with mechanisms adopted by other oncoviruses (Mattoscio et al., 2018).

## Diagnosis

### Cytology and Histology

The commencement of screening programs over the last several decades has achieved its goal of lowering the occurrence and mortality of cervical cancer (Martinhirsch and Wood, 2011). The Papanicolaou smear is the most frequently used and easiest method to evaluate the cervix for dysplastic cellular modifications. During the procedure, the vagina is held open with a speculum, and a specimen of cells is obtained from the squamocolumnar junction by inserting and rotating a spatula through the external os. The smear is fixed onto a slide, properly stained, and viewed under a microscope to see the morphology of the epithelial cells. A colposcope enables the immediate observation of the cervix with magnified epithelium and the ability to perform a biopsy (Cubie, 2013). It has been estimated that 40–50% of cervical cancers are diagnosed in women who undergo routine cervical cytology screening and that four out of five women diagnosed with cancer had not been tested in the previous 5 years (Cubie, 2013).

Liquid-based cytology has enabled the optimization of the quality and regularity of specimens by producing identical cell layers and lowering the amount of low-quality specimens. The existence of koilocytes is typical of a productive HPV infection, whereas a consistent infection reveals itself as causing extreme alterations in the nucleus, mitotic figures, and aggregates of pyknotic cells. Various classification techniques are utilized for these observations (see Table 1), but the Bethesda classification is typically the one that is selected and offers two categories: (1) low-grade squamous epithelial neoplasia (LSIL) that surrounds specimens with asymmetrical, large cells and well-defined nuclei and (2) high-grade squamous epithelial neoplasia (HSIL) for specimens identified by poorly differentiated and underdeveloped, small cells with definitive cytoplasmic borders that are arranged in sheets and syncytial groups (Cubie, 2013).

**Table 1:** Classification of cervical intraepithelial lesions.

Papanicolaou classification	Dysplasia classification	Bethesda classification	Histology classification
I	Negative squamous atypia	NILM (negative for intraepithelial lesion or malignancy)	Negative
II	Squamous atypia	ASCUS (atypical squamous cell of unknown significance), ASC-H (atypical squamous cells – cannot exclude HSIL)	Squamous atypia
	Mild	LSIL (Low grade squamous intra-epithelial lesions)	CIN1 (abnormal cells in one of the three layers; very unlikely to progress)
II	Moderate	HSIL (high grade squamous intraepithelial lesions)	CIN 2 (Abnormal cells including mitotic figures in two of the three layers with loss of stratification and differentiation) CIN 3 (Abnormal cells in all layers; can progress to invasive cancer if untreated)
IV	Severe CIS (carcinoma *in situ*)	HSIL	CIN 3 (abnormal cells in all layers; can progress to invasive cancer if untreated)
V	Carcinoma	Carcinoma	Carcinoma

The histological assessment of samples offers more precise data regarding the HPV infection by detailing characteristics that include basal hypertrophy, a surplus of surface keratinization, and the overall disturbance of the epithelium. Cervical intraneoplasia lesions are graded according to the fraction of epithelium that exhibits abnormalities (Cubie, 2013).

### Biomarkers

There are different biomarkers that can determine the existence and level of HPV infection and degree of cervical malignancy. The biomarkers can be separated into three groups. The first one contains HPV DNA, RNA, and proteins, which are highly sensitive and specific to diagnose CIN 2 or more extensive lesions in women who are 30 years of age or older and to identify adenocarcinoma. The second one includes cellular biomarkers that are associated with E6 and E7 proteins changing some pathways that regulate the cell cycle. For example, since the silencing of pRb produces a rise in the CDK inhibitor p16, the overexpression of this molecule can be determined by immunostaining or enzyme-linked immunosorbent assay (ELISA) and thus serves as a marker of cellular transformation. The third group contains epigenetic biomarkers that reveal DNA methylation and show if DNA has been activated or silenced. The state of methylation of the L1 gene appears to be associated with the diagnosis of CIN 2 (Tornesello et al., 2013). Table 2 summarizes the different types of biomarkers used in the detection of cervical cancer lesions.

**Table 2:** HPV infection biomarkers.

Type of biomarker	Test/Technique	Remarks
HPV DNA testing	Hybrid capture 2 (Qiagen) detects 13 high-risk HPVs	Detects 13 high-risk HPVs
	Cervista HPV HR (Hologic) detects 14 high-risk HPVs	Detects 14 high-risk HPVs
	Cervista HPV 16/18 (Hologic)	Specifically identifies HPV 16 and 18
	Cobas 4800 HPV (Roche diagnostics)	Targets 14 high-risk HPVs
HPV RNA testing	APITMA (Gen-Probe) and OncoTect (IncellDX)	Based on reverse transcriptase and PCR technique. Can detect E6 and E7 mRNA from 14 and 13 high-risk HPA serotypes, respectively
	PreTect HPV-Proofer (Norchip) and NucliSENS EasyQ (bioMerieux)	Rely on nucleic acid sequence-based amplification (NAS-BA) and are able to detect E6/E7 transcripts from HPV 16, 18, 31, 33, and 45
HPV protein testing	OncoE6 (Arbor Vita Corporation)	Detects E6 protein encoded by HPV 16, 18, and 45
	Cytoactiv assay (cytoimmune diagnostics)	Measures loss of expression of L1 which has been identified as a potential marker of progressive lesions
Cellular biomarkers	P16/K1-67 immunocytochemistry assay	p16 is a CDK-I while K1-67 is a proliferation antigen expressed in the G2 and M phases of the cell cycle. They are co-expressed in dysplastic lesions and constitute a highly sensitive and specific test for CIN 2 or worse lesions
	ProExCTM assay (Becton-Dickinson)	Recognizes minichromosome maintenance protein 2 and topoisomerase II α, which are expressed in cells with abnormal S-phases such as HPV-infected cells with increased E6/E7 synthesis.
	Fluorescence *in situ* hybridization (FISH) and multiplex ligation-dependent probe amplification (MLPA)	Can be used to detect gain of chromosomes 3q and 5p which carry the TERC and TERT genes
Epigenetic biomarkers	Differential methylation hybridization (DMH)	Allows identification of SOX1, NKX6-1, PAX1, WX1, and LMXIA genes that are often methylated in cervical cancer and precancerous lesions
	Restriction landmark genomic scanning (RLGS)	Detection of methylated NOL4 and LHFPL4 genes
	Demethylating agent expression microarray	Identifies methylation of SPARC and TFP2 genes

## Management

Cervical cancer prognosis and the proper treatment for the condition are based on the stage of the cancer, which is decided by the level of invasion and how far the tumor has extended. While cervical cancer is staged based on International Federation of Obstetrics and Gynecology guidelines, it is classified into three sub-groups for treatment (Duenasgonzalez et al., 2014). The earliest stages (IA_1_–IIA_1_) show tumors on the upper 1/3 of the vagina that measure <4 cm and are treated via conization with sufficient excision margins for IA_1_ and conization and simple/radical hysterectomy with pelvic lymphadenectomy for IIA_2_. Patients with small-volume macroscopic disease (IIA_1_–IB_1_) receive radical hysterectomies, while radical trachelectomy with lymphadenectomy is utilized to retain fertility (Martinhirsch and Wood, 2011). Intermediate stages (IB_2_–IVA) require primary chemoradiation (Duenasgonzalez et al., 2014), whereas advanced stages (IVB) and those with ongoing incurable disease are given systemic chemotherapy, including cisplatin, carboplatin, paclitaxel, topotecan, and gemcitabine (Scatchard et al., 2012).

Research is now closing in on novel molecular targets for cervical cancer therapeutics, including the utilization of epidermal growth factor receptor (EGFR) antagonists, such as panitumumab, and multitargeted tyrosine kinase inhibitors, such as imatinib and sunitinib (Candelaria et al., 2009; Duenasgonzalez et al., 2014). In addition, epigenetic therapy seems to be a potential and efficient type of therapy for cervical cancer (Dueñasgonzález et al., 2005). Investigations on the effects of adding the antiangiogenesis agent bevacizumab to chemotherapy used in advanced or recurrent disease are also being carried out (Tewari et al., 2014).

## Prevention

The HPV vaccine is a vital method of protection against cervical cancer, particularly when combined with a healthy sexual lifestyle and the proper use of contraceptives. There has been a rise in backing the vaccination of men against HPV; it is a practical choice for two reasons. Since HPV serotypes 16 and 18 are connected to 70% of anal cancers and precancerous lesions of the penis, the HPV vaccine can limit the occurrence of these conditions, particularly in homosexual men who are at a high risk of anal dysplasia. The vaccination is also pertinent for heterosexuals who participate in anal intercourse. It has been determined that 35.9% of women and 42.3% of men 18–44 years of age have anal intercourse with people of the opposite sex (Copen et al., 2016). In addition, vaccination of men is also a secondary way to protect against HPV-induced cervical cancer in women. Regrettably, populations in low-income countries are not as likely to receive the HPV vaccination due to its high cost. Further, the majority of women in these areas are not able to be regularly screened, although Pap smear screening has increased approximately 5% during the last 5 years (Katz and Wright, 2006). The low rates of screening result in an overall higher mortality rate from cervical cancer in developing countries.

### HPV Vaccines

There are currently two available vaccines against HPV, Gardasil^®^ made by Merck Frost, and Cervarix^®^ made by GlaxoSmithKline. Both vaccines contain virus-like particles (VLPs) produced with a recombinant DNA platform. They both have the L1 protein portion of the viral capsids (Katz and Wright, 2006). These types of VLPs generate strong reactions from the immune system and produce antibody titers that are much greater than those prompted by natural infection (Dawar et al., 2007).

### Quadrivalent Vaccine

Gardasil^®^ is a quadrivalent vaccine because it targets four HPV serotypes, namely 6, 11, 16, and 18. The L1 capsid proteins are produced in yeast cells (*Saccharomyces cerevisiae*) and mixed with an aluminum adjuvant. The vaccine is suggested for both males and females who are 9–26 years of age, and it is given in three doses at 0, 2, and 6 months. Gardasil^®^ can safeguard against continuous HPV infection, precancerous lesions of the cervix, vulva, and vagina, and genital warts linked to serotypes 11, 16, and 18 in females who are 16–26 years of age who have not experienced a prior infection (Mello, 2013).

### Bivalent Vaccine

Cervarix^®^ is a bivalent vaccine that provides protection against the two serotypes HPV 16 and 18. The VLPs in this preparation are generated in insect cells with baculovirus and an adjuvant with ASO_4_ and monophosphoryl lipid A via bacterial cell walls (Dawar et al., 2007; Mello, 2013). The vaccine is designed for girls and women 10–25 years of age and is given in three doses at 0, 1, and 6 months. Randomized, controlled, and double-blind studies with women between 15 and 25 years of age showed that the vaccine is effective in averting precursor lesions of cervical cancer associated with HPV 16 and 18 in women without prior infection (Mello, 2013).

Upon viewing the variation of antibody titer with the amount of time following the administration of the Gardasil^®^ vaccine, it is important to recognize that the variation in titer values after the Cervarix^®^ vaccine has an identical profile, with the exception of two variations. First, at 24 months following the vaccination with Gardasil^®^, 96% of the patients were positive for HPV types 6, 11, and 16 antibodies, and 68% tested positive for type 18. Seropositivity is achieved in 100% of patients for HPV-16 and HPV-18 antibodies at 51–53 months following the vaccination. Second, the plateau achieved with Gardasil^®^ is close to the titers that are spontaneously prompted by types 6 and 18 and is increased for types 11 and 16, while the plateau of Cervarix^®^ is greater than that induced by the naturally occurring infection (Dawar et al., 2007).

### Nine-Valent Vaccine

The non-avalent vaccine, which represents a new vaccine generation, has been recently approved by Merck EMA (Eisele et al., 2013; Joura et al., 2015; Schiller and Müller, 2015). The new vaccine increases the coverage from the four original types included in Gardasil^®^ to five more oncogenic types (HPV 31, 33, 45, 52, and 58). Coverage of the additional types may increase type-specific protection from approximately 70 to 90% of cervical cancer-causing HPV infections. In 2013, Merck announced that they had achieved the pre-specified endpoints of their large phase three efficacy trial (Herrero et al., 2015). When compared with the quadrivalent vaccine, the non-avalent vaccine was non-inferior in inducing antibodies to the four shared VLPs and reduced the moderate and high-grade cervical dysplasia attributable to the five additional VLP types by 96%. The seven oncogenic types included in the new vaccine (HPV 16, 18, 31, 33, 45, 52, and 58) cause most HPV infections that quickly progress to high-grade precursors of cervical cancer. Eventually, the non-avalent vaccine could lead to longer intervals between screenings for cervical cancer in women who have been vaccinated against HPV, resulting in significant cost savings to cervical cancer prevention programs (Printz, 2015; Schiller and Müller, 2015).

### Vaccine Efficacy

The bivalent vaccine was evaluated in a phase III study sponsored by GlaxoSmithKline called the Papilloma Trial Against Cancer in Young Adults (Paavonen et al., 2009; Luckett and Feldman, 2016; St Laurent et al., 2018). In this multinational prospective double-blind placebo-controlled trial, the vaccine was 92.9% effective in preventing CIN 2 in 18,000 women ages 15–25 with either normal cervical cytology or low grade cervical dysplasia (Table 3). The vaccine was 52.8% effective in preventing these lesions as well as 33.6% effective in preventing high risk CIN 3 lesions in patients with a history of HPV infection (Table 3).

**Table 3:** Efficacy of HPV vaccines available.

		HPV vaccine type (HPV types included)		
		Bivalent (HPV16, 18)	Quadrivalent (6, 11, 16, 18)	Non-avalent (6, 11, 16, 18 31, 33, 45, 52, and 58)
Efficacy in HPV-naïve women	Prevention of vaccine-specific HPV type infection	94.3% (HPV16/18) at 3.6 year 87.9% (HPV16/18) at 4 year	97% (HPV16) at 3.7 year	96% (HPV16/18) at 4.5 year 100% (HPV18) at 3.7 year
	Prevention of CIN 2+++ associated with vaccine-specific HPV	92.9% at 3.6 year	100% (CIN 2) at 3.7 year 97% (CIN 3) at 3.7 year	96.3% at 4.5 year
	Prevention of CIN 2+ associated with any HPV type	61.9% at 3.6 year	No data	No data
	Prevention of anal HPV associated with any HPV type	83.6% at 4.0 year	No data	No data
Efficacy in all women (including HPV-exposed)	Prevention of vaccine-specific HPV type infection	76.4% at 4 year	42% (HPV16) at 3.7 year 79% (HPV18) at 3.7 year	80.2% at 4.5 year


	Prevention of CIN 2+ associated with vaccine-specific HPV	52.8% (CIN 2+) at 3.6 year 33.6% (CIN 3+) at 3.6 year	57% (CIN 2) at 3.7 year 45% (CIN 3) at 3.7 year	No data
	Prevention of CIN 2+ associated with any HPV type	30.4% (CIN 2+) at 3.6 year 33.4% (CIN 3+) at 3.6 year	17% (CIN 2+) at 3.7 year	No data
	Prevention of Anal HPV associated with any HPV type	62.0% at 4.0 year	No data	No data
Efficacy in HPV-naïve men	Prevention of vaccine-specific HPV type infection	No data	47.8% at 2.9 year	No data
	Prevention of anogenital lesions	No data	90.4% at 2.9 year	No data
Efficacy in all men (including HPV-exposed)	Prevention of vaccine-specific HPV type infection	No data	27.1% at 2.9 year	No data
	Prevention of anogenital lesions	No data	65.5% at 2.9 year	No data
Reference		Luckett and Feldman, 2016; St Laurent et al., 2018	FIS Group, 2007; St Laurent et al., 2018	Luckett and Feldman, 2016; St Laurent et al., 2018

The qHPV vaccine was evaluated in a series of Merck sponsored trials: Females United to Unilaterally Reduce Endo/Ectocervical Disease (FUTURE) trials (FIS Group, 2007; Garland et al., 2007). In the first smaller trial, qHPV vaccine was 100% effective against CIN 2/3 in HPV naive women (Table 3). The follow-up trial, FUTURE II, was a larger phase III double-blinded, multinational prospective, placebo-controlled trial of over 12,000 women. Qualified study subjects were between the ages of 15–26 without a history of abnormal Papanicolaou smears and had less than four lifetime sexual partners. In this group, qHPV vaccine was 98% efficacious against a composite of CIN 2/3, HPV 16/18 infection, as well as adenocarcinoma *in situ* (AIS) in women ages 15 to 26. Additionally, the vaccine was 42% effective against HPV 16 and 79% effective against HPV 18 (Table 3). In the same group, qHPV vaccine was, respectively, 57 and 45% effective against HPV 16 and 18 associated CIN 2/3, preventing approximately 17% of all CIN 2 lesions (Table 3). Importantly, the qHPV vaccine demonstrated limited cross-protection, 32.5%, against CIN 2/3 and AIS in the FUTURE trials (Table 3). The efficacy of the 9vHPV vaccine was evaluated in a phase IIb-III double-blinded, randomized international non-inferiority trial supported by Merck (Luckett and Feldman, 2016; St Laurent et al., 2018). In 14,215 low risk women ages 16–26 randomized to a 3-dose regimen of either 9vHPV vaccine or qHPV, the 9vHPV vaccine was 96% effective at preventing high-grade CIN, AIS, high-grade VAIN, vulvar cancer, and vaginal cancer associated with the nine HPV subtypes targeted by the 9vHPV vaccine. 9vHPV was 96% effective at preventing persistent infections related to the same HPV-types. However, analysis of women including those with prior HPV infection showed no difference in cervical, vaginal, or vulvar disease between the two vaccines (Luckett and Feldman, 2016).

### Contraindications and Side Effects

The vaccine is contraindicated in patients with allergies to any of its components, and, while it does not seem to escalate the occurrence of miscarriage, pregnant women should not receive the vaccination (Schiller et al., 2012; Mello, 2013; Schiller and Müller, 2015). Additionally, patients with direct hypersensitivity to yeast cannot be Gardasil^®^ vaccine recipients. The vaccine is not contraindicated for patients with immunosuppressive conditions or patients with prior HPV infections, but immunogenicity cannot be assured for the former group. Additional vaccines can be given prior to, during, and following the HPV vaccination (Mello, 2013). Side effects that have been reported for the quadrivalent vaccine are: (1) pain, swelling, and redness at the injection site and headache in more than 10% of recipients; (2) bruising, itching, fever, and nausea in more than 0.1%; (3) urticaria in less than 0.1%; and (4) bronchospasm in less than 0.01% (Kitchener et al., 2006; Burd, 2016).

## Future Challenges

Despite the development of preventive vaccines that target high-risk HPV types, their usage rates remain low. Increased public health efforts are required to improve HPV vaccine usage in the United States. In the case of less-developed countries in which the number of cervical cancer cases is high, single-dose vaccines need to be developed. Furthermore, basic scientific research is required to aid in understanding the factors that regulate the development of cancer after HPV infection. Discovery of the factors that mediate this progression, such as disruption of signaling, pathway differentiation, and changes in epigenetic chromatin dynamics, will provide significant insight into the mechanisms of development of other cancers. In the future, research efforts will be most advantageous when broken up into three areas (Langsfeld and Laimin, 2016): (i) understanding how HPV infections develop into cancer, specifically, how the viruses communicate with host chromatin remodeling processes, DNA repair, and differentiation pathways; (ii) examining of HPV infections and oropharynx cancers, since they appear to be different from cancers of the anogenital tract; and (iii) evaluating the effects of immunomodulation in HPV infection and the development of immune therapies for women currently infected with HPV. We believe that the exploration of these aspects is of great urgency for HPV research in the next 10 years.

Medical developments will continue; novel tests, markers, and evidence will increase our capability to distinguish between minor HPV infections and precancerous and cancerous disease. The main goal is to have an easy, strong, and cost-efficient system in place that will provide better patient care.

## Author Contributions

YZ and XH provided ideas and chaired the projects. XW and YZ designed the research and were associated with data analysis. XW performed the other works and wrote the manuscript.

## Conflict of Interest Statement

The authors declare that the research was conducted in the absence of any commercial or financial relationships that could be construed as a potential conflict of interest.
